# Influence of Gradual Elongation to the Patella Tendon Insertion in Rabbits

**DOI:** 10.3390/ijms150814835

**Published:** 2014-08-22

**Authors:** Hirotaka Mutsuzaki, Hiromi Nakajima, Yasuyoshi Wadano, Shintarou Watanabe, Masataka Sakane

**Affiliations:** 1 Department of Orthopaedic Surgery, Ibaraki Prefectural University of Health Sciences, 4669-2 Ami Ami-machi, Inashiki-gun, Ibaraki 300-0394, Japan; 2 Department of Agriculture, Ibaraki University, 3-21-1 Chuuou, Ami-machi, Inashiki-gun, Ibaraki 300-0393, Japan; E-Mails: hiromi@mx.ibaraki.ac.jp (H.N.); hiromi@mx.ibaraki.ac.jp (S.W.); 3 Department of Rehabilitation Medicine, Ibaraki Prefectural University of Health Sciences, 4669-2 Ami Ami-machi, Inashiki-gun, Ibaraki 300-0394, Japan; E-Mail: wadano@ipu.ac.jp; 4 Department of Orthopaedic Surgery, University of Tsukuba, 1-1-1 Tennoudai, Tsukuba, Ibaraki 305-8575, Japan; E-Mail: sakane-m@md.tsukuba.ac.jp

**Keywords:** insertion, chondrocytes apoptosis, chondrocyte proliferation, glycosaminoglycan, gradual elongation, external fixation

## Abstract

The purpose of this study was to examine the histological changes at the patella tendon (PT) insertion site under gradual elongation in rabbits. Gradual elongation of the PT was performed using external fixation for 4 weeks, with a lengthening speed of 0.5 mm/day (elongation group; *n* = 24). Rabbits in the sham group underwent the same surgical procedure without gradual elongation (sham group; *n* = 24). Eight animals were sacrificed 1, 2 and 4 weeks after surgery in each group, respectively. Average thicknesses of stained glycosaminoglycan (GAGs) areas by Safranin-O staining in the total cartilage layer and the uncalcified fibrocartilage layer in the elongation group were significantly higher than that in the sham group at 4 weeks (*p* < 0.05) and that in the intact PT group (*n* = 6, *p* < 0.05). In the elongation group, the peak in the average thicknesses of the stained GAGs areas in the total cartilage layer and the uncalcified fibrocartilage layer were observed at 4 weeks. Gradual elongation of PT insertion significantly affected the increase in the average thicknesses of the stained GAGs areas in the cartilage layer especially in the uncalcified fibrocartilage layer at 4 weeks in rabbits. Clinically, insertions of tendon and ligament can extend during gradual elongation using external fixation more than 4 weeks after the operation.

## 1. Introduction

A tendon/ligament insertion site, such as that of the patella tendon (PT) or the anterior cruciate ligament (ACL), represents a functionally graded material system that exhibits a gradual transition in the tissue from an unmineralized tissue (*i.e.*, tendon or ligament), to an unmineralized fibrocartilage, to a mineralized fibrocartilage, and finally to a mineralized tissue (*i.e.*, bone) [1,2,3]. The gradual increase in hardness of each of the four tissues reduces stress concentration at the insertion site [1,3]. In addition, the associated gradation in mechanical properties of the insertion site tissue is also believed to ameliorate stress, facilitating the safe transmission of force. Furthermore, the glycosaminoglycans (GAGs) and cartilage layer are presumed to resist tensile, compressive and shear stresses at the insertion site [3] and provide the ability for the tissue to absorb water and maintain flexibility in the ligaments [4]. 

In humans, ACL tibial insertions after rupture result in an increase in the chondrocyte apoptosis rate and a decrease in average thicknesses of the stained GAG areas; this is also well replicated in ACL tibial insertions after ACL resection in rabbit models of repair in our previous studies [5,6,7,8]. On the other hand, in the ACL partial resection animal model, the average thicknesses of the stained GAGs areas in the remaining ligament area increased up until 4 weeks, and then gradually decreased until 8 weeks owing to an imbalance between chondrocyte apoptosis and chondrocyte proliferation [9]. Therefore, it appears that an insertion undergoes morphological and structural changes under the guidance of the mechanical stress it receives from the ligaments. 

Bone lengthening using external fixation is widely performed as a treatment for congenital deformities and limb length discrepancy caused by trauma [10,11,12]. During bone lengthening, soft tissues such as the skin, muscles, nerves, and blood vessels are passively elongated, with a lengthening speed of 0.5–1 mm/day of gradual elongation deemed appropriate as a passive tensile stress for the living body [10,11,12].

We hypothesized that an increase in average thicknesses of the stained GAGs areas would be associated with gradual elongation of PT insertion in rabbits. This is the first study to clarify the responses of chondrocytes and changes in the average thicknesses of the stained GAGs areas in a PT insertion under gradual elongation. The purpose of this study was to examine the histological changes in PT insertion under gradual elongation using external fixation model in rabbits.

## 2. Results

### 2.1. Proportion of TUNEL-Positive Chondrocytes (Figure 1a and Figure 2)

TUNEL were performed to detect apoptosis. The average percentages of TUNEL-positive chondrocytes in the sham group (20.22% ± 11.00%) was significantly higher than that in the elongation group at 2 weeks (8.78% ± 9.29%; *p* = 0.025) and that in the intact PT group (9.39% ± 5.26%; *p* = 0.023). In the elongation group, no significant differences were found in the average percentages of TUNEL-positive chondrocytes between any pair of the 1, 2, and 4 weeks. In the sham group, the average percentages of TUNEL-positive chondrocytes at 2 weeks (20.22% ± 11.00%) was significantly higher than that at both 1 week (7.24% ± 5.58%; *p* = 0.007) and at 4 weeks (6.18% ± 4.70%; *p* = 0.047).

**Figure 1:**
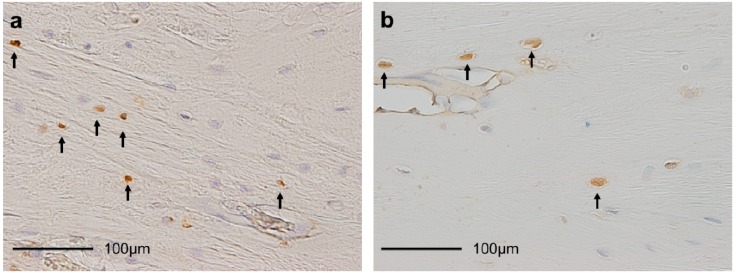
Histological sections of the patella-tendon (PT) insertions (×400). (**a**) TUNEL staining. Brown cells (arrows) are TUNEL-positive chondrocytes; and (**b**) Proportion of proliferating cell nuclear antigen (PCNA) staining. Brown cells (arrows) are PCNA-positive chondrocytes.

### 2.2. Proportion of Proliferating Cell Nuclear Antigen (PCNA)-Positive Chondrocytes (Figure 1b and Figure 3)

The average percentage of proportion of proliferating cell nuclear antigen (PCNA)-positive chondrocytes in the elongation group (20.37% ± 10.35%) was significantly higher than that in the sham group at 4 weeks (10.63% ± 4.72%; *p* = 0.018). In the elongation group, no significant differences were found in the average percentages of PCNA-positive chondrocytes between any pair of the 1, 2, and 4 weeks. In the sham group, the average percentages of PCNA-positive chondrocytes at 2 weeks (18.94% ± 9.42%) was higher than that at both 1 week (11.55% ± 5.31%; *p* = 0.037) and at 4 weeks (10.63% ± 4.72%; *p* = 0.025). The average percentage of PCNA-positive chondrocytes in the intact PT group was 15.24% ± 10.64%. No significant differences were found in the average percentage of PCNA-positive chondrocytes between any pair of the intact PT, the sham group (1, 2 and 4 weeks), and the elongation groups (1, 2 and 4 weeks) (*p* = 0.146–0.467).

**Figure 2:**
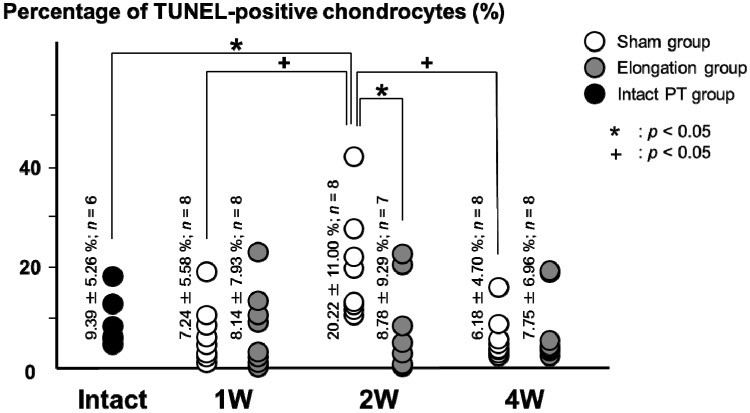
Proportion of TUNEL-positive cells. The average percentages of TUNEL-positive chondrocytes in the sham group was significantly higher than that in the elongation group at 2 weeks (*p* = 0.025) and that in the intact PT group (*p* = 0.023). In the sham group, the average percentages of TUNEL-positive chondrocytes was significantly higher at 2 weeks as compared with 1 week (*p* = 0.007) and 4 weeks (*p* = 0.047).

**Figure 3:**
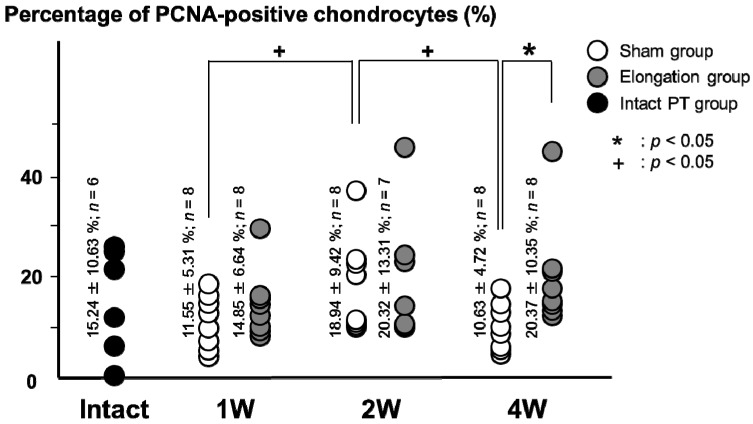
Proportion of PCNA-positive cells. The average percentages of PCNA-positive chondrocytes in the elongation group was significantly higher than that in the sham group at 4 weeks (*p* = 0.018). In sham group, the average percentages of PCNA-positive chondrocytes at 2 weeks is higher than those at 1 week (*p* = 0.037) and 4 weeks (*p* = 0.025).

### 2.3. Average Thickness of the Stained GAGs Areas by Safranin-O Staining

#### 2.3.1. Total Cartilage Layer (Figure 4 and Figure 5)

The average thicknesses of the stained GAGs areas in the elongation group at 4 weeks (64.34 ± 33.20 μm) was significantly higher than that in the sham group at 4 weeks (26.94 ± 20.52 μm; *p* = 0.008). In the elongation group, the average thicknesses of the stained GAGs areas at 2 weeks (22.79 ± 11.13 μm) and 4 weeks (64.34 ± 33.20 μm) were higher than that at 1 week (12.05% ± 8.17%; *p* = 0.025 and *p* < 0.001, respectively). In the elongation group, the average thickness of the stained GAGs areas at 4 weeks (64.34 ± 33.20 μm) was higher than that at 2 weeks (22.79 ± 11.13 μm; *p* = 0.004). The average thickness of the stained GAGs areas in the elongation group at 4 weeks (64.34 ± 33.20 μm) was significantly higher than that in the intact PT group (32.37 ± 14.54 μm; *p* = 0.025). The average thicknesses of the stained GAGs areas in the elongation group at 1 week and the sham group at 1 and 2 weeks were significantly lower than that in the intact PT group (*p* = 0.003, 0.014 and 0.006, respectively). In the sham group, no significant differences were found in the average thicknesses of the stained GAGs areas between any pair of the 1, 2, and 4 weeks.

**Figure 4:**
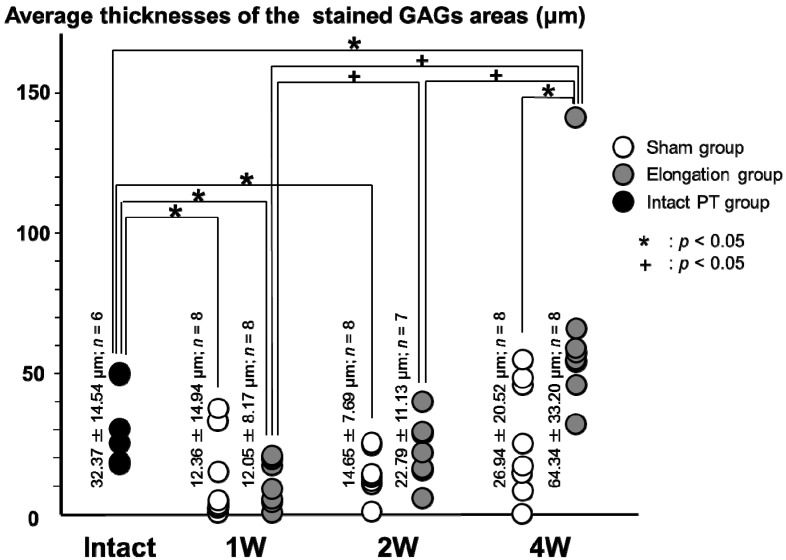
Average thicknesses of the stained glycosaminoglycans (GAGs) areas in the total cartilage layer. The average thickness of the stained GAGs areas in the elongation group at 4 weeks was significantly higher than that in the sham group at 4 weeks (*p* = 0.008). In the elongation group, the average thicknesses of the stained GAGs areas at 2 and 4 weeks are higher than that at 1 week (*p* = 0.025 and *p* < 0.001, respectively). In the elongation group, the average thickness of the stained GAGs areas at 4 weeks was higher than that at 2 weeks (*p* = 0.004). The average thickness of the stained GAGs areas in the elongation group at 4 weeks was significantly higher than that in the intact PT group (*p* = 0.025). The average thicknesses of the stained GAGs areas in the elongation group at 1 week and the sham group at 1 and 2 weeks were significantly lower than that in the intact PT group (*p* = 0.003, 0.014 and 0.006, respectively).

**Figure 5:**
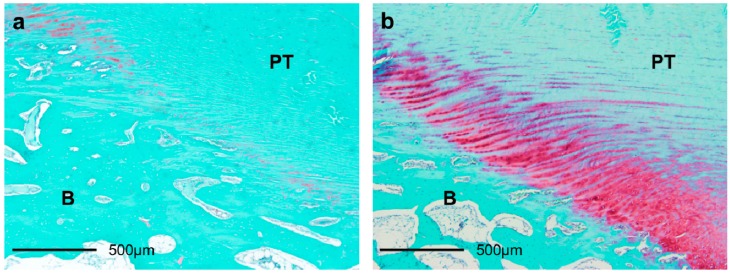
Safranin-O staining of histological sections of PT insertions 4 weeks after surgery (×40). The thickness of the stained GAGs area (stained red) in the elongation group (**b**) was greater than that in the sham group (**a**). PT: patella tendon. B: bone.

**Figure 6:**
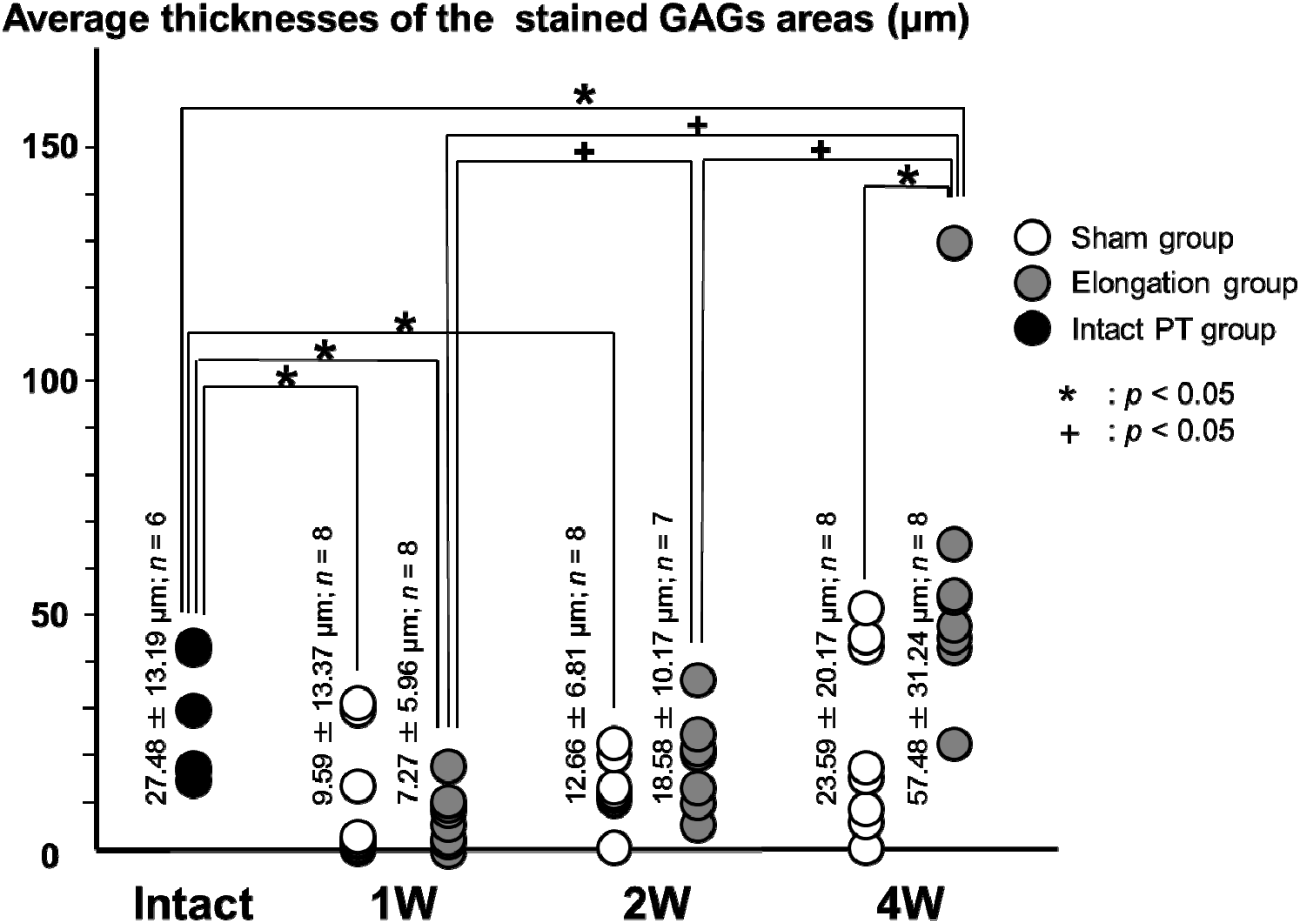
Average thicknesses of the stained GAGs areas in the uncalcified fibrocartilage layer. The average thickness of the stained GAGs areas in the elongation group at 4 weeks was significantly higher than that in the sham group at 4 weeks (*p* = 0.011). In the elongation group, the average thicknesses of the stained GAGs areas at 2 and 4 weeks were higher than that at 1 week (*p* = 0.009 and *p* < 0.001, respectively). In the elongation group, the average thickness of the stained GAGs areas at 4 weeks was higher than that at 2 weeks (*p* = 0.004). The average thickness of the stained GAGs areas in the elongation group at 4 weeks was significantly higher than that in the intact PT group (*p* = 0.024). The average thicknesses of the stained GAGs areas in the elongation group at 1 week and the sham group at 1 and 2 weeks was significantly lower than that in the intact PT group (*p* = 0.001, 0.014 and 0.009, respectively).

#### 2.3.2. Uncalcified Fibrocartilage Layer (Figure 6)

The average thicknesses of the stained GAGs areas in the elongation group at 4 weeks (57.48 ± 31.24 μm) was significantly higher than that in the sham group at 4 weeks (23.59 ± 20.17 μm; *p* = 0.011). In the elongation group, the average thicknesses of the stained GAGs areas at 2 weeks (18.58 ± 10.17 μm) and 4 weeks (57.48 ± 31.24 μm) were higher than that at 1 week (7.27 ± 5.96 μm; *p* = 0.009 and *p* < 0.001, respectively). In the elongation group, the average thickness of the stained GAGs areas at 4 weeks (57.48 ± 31.24 μm) was higher than that at 2 weeks (18.58 ± 10.17 μm; *p* = 0.004). The average thickness of the stained GAGs areas in the elongation group at 4 weeks (57.48 ± 31.24 μm) was significantly higher than that in the intact PT group (27.48 ± 13.19 μm; *p* = 0.024). The average thicknesses of the stained GAGs areas in the elongation group at 1 week and the sham group at 1 and 2 weeks were significantly lower than that in the intact PT group (*p* = 0.001, 0.014 and 0.009, respectively).In the sham group, no significant differences were found in the average thicknesses of the stained GAGs areas between any pair at 1, 2, and 4 weeks. 

**Figure 7:**
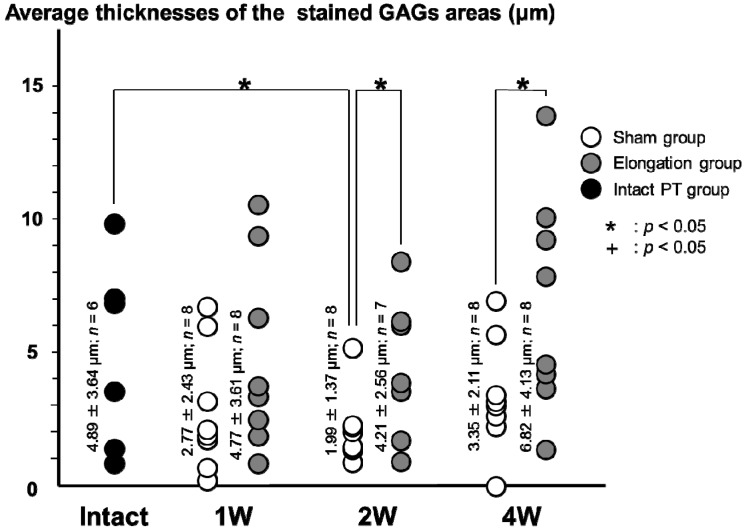
Average thickness of the stained GAGs areas in the calcified fibrocartilage layer. The average thicknesses of the stained GAGs areas in the elongation group was significantly higher than that in the sham group at both 2 weeks (*p* = 0.026) and 4 weeks (*p* = 0.027). In the sham group and the elongation group, no significant differences were found in the average thicknesses of the stained GAGs areas between any pair at 1, 2, and 4 weeks. The average thickness of the stained GAGs areas in the sham group at 2 weeks was significantly lower than that in the intact PT group (*p* = 0.029).

#### 2.3.3. Calcified Fibrocartilage Layer (Figure 7)

The average thickness of the stained GAGs areas in the elongation group was significantly higher than that in the sham group at both 2 weeks (4.21 ± 2.56 * vs.* 1.99 ± 1.37 μm; *p* = 0.026) and 4 weeks (6.82 ± 4.13 *vs.* 3.35 ± 2.11 μm; *p* = 0.027). In the sham group and the elongation group, no significant differences were found in the average thicknesses of the stained GAGs areas between any pair at 1, 2, and 4 weeks. The average thickness of the stained GAGs areas in the sham group at 2 weeks (1.99 ± 1.37 μm) was significantly lower than that in the intact PT group (4.89 ± 3.64 μm; *p* = 0.029).

## 3. Discussion

Following the application of the passive tensile stress under gradual elongation in PT insertion using external fixation, the increase in the average thicknesses of the stained GAGs areas in the cartilage layer especially in the uncalcified fibrocartilage layer was observed 4 weeks after the operation.

In the elongation group, the increase in the average thicknesses of the stained GAGs areas in the cartilage layer especially in the uncalcified fibrocartilage layer was observed at 4 weeks compared with the sham group and the intact PT group. In our previous animal experiments using an ACL partial resection model, average thicknesses of the stained GAGs areas increased at 2–4 weeks in the remaining ligament area [9]. Moreover, the reactions of uncalcified fibrocartilage layer preceded those of calcified fibrocartilage layer in the animal experiment of the ACL complete resection and partial resection [8,9]. We considered that the PT insertion of the elongation group could withstand larger tensile stresses than those of the sham group and the intact PT group. The uncalcified fibrocartilage layer in the PT insertion may be affected by the passive tensile stress compared with the calcified fibrocartilage layer up to 4 weeks. The differences in the histological changes between the uncalcified fibrocartilage layer and the calcified fibrocartilage layer might be due to differences of tissue hardness, differences in the transmission order of mechanical stress. The average thickness of the stained GAG areas in the calcified fibrocartilage layer in the elongation group at 4 weeks was significantly higher than that in the sham group. However, no significant differences were found in the average thicknesses of the stained GAG areas in the calcified fibrocartilage layer between the elongation group at 4 weeks and the intact PT group. It is necessary to perform long-term experiments exceeding 4 weeks to clarify the increase in the average thicknesses of the stained GAG areas in the calcified fibrocartilage layer. The regulation of cell behaviour via the mechanical environment, especially tensile stress, is an important factor at the insertion site. Indeed, studies show that tensile stress applied to mesenchymal stem cells (MSCs) *in vitro* results in increases in tendon-related matrix deposition and cell alignment [13]. MSC-seeded collagen type I sponges were loaded in cyclic tension for up to 2 weeks. Compared with unstimulated constructs, mechanically loaded constructs exhibited 3–4 times greater collagen type gene expression [14]. Fibrocartilage generation was investigated with multipotent cells by observing how biochemical and mechanical cues in MSCs together modulate differential and phenotypic behaviour over a 7-day culture period in a 3D collagen matrix [15]. Sox9 was upregulated under static loading conditions and aggrecan was upregulated under cyclic loading conditions.

In the elongation group, the PCNA-positive chondrocytes rate in the elongation group was significantly higher than that in the sham group at 4 weeks. However, no significant differences were found in the PCNA-positive chondrocytes rate between the elongation group at 4 weeks and the intact PT group. The reaction in the elongation group at 4 weeks may be a result of the passive tensile stress and/or the recovery from inflammation of the surgery. In our previous animal experiments using an ACL partial resection model, the PCNA-positive chondrocytes rate increased at 2–4 weeks in the remaining ligament area [9]. Proliferation of the growth plate chondrocytes was observed under gradual physeal distraction by an external fixation device in rabbits [16]. It is necessary to perform long-term experiments exceeding 4 weeks including comparison with the intact PT group.

In the sham group, the peak in chondrocyte apoptosis and chondrocyte proliferation were observed at 2 weeks. The chondrocyte apoptosis rate at 2 weeks in the sham group was significantly higher than that in the elongation group at 2 weeks and the intact PT group. The average thicknesses of the stained GAGs areas in the total cartilage layer and the uncalcified fibrocartilage layer at 1 and 2 weeks in the sham group and 1 week in the elongation group were significantly lower than that in the intact PT group. The average thicknesses of the stained GAGs areas in the calcified fibrocartilage layer at 2 weeks in the sham group were significantly lower than that in the intact PT group. The authors suggest that the histological changes may occur in association with proinflammatory responses of the surgery at 1 and 2 weeks in both groups. In the acute and subacute phases after injury, inflammatory cytokines are considered to affect chondrocyte apoptosis and matrix degradation [17,18].

Tendon- or ligament-to-bone attachments are injured routinely in response to localized stresses, and these sites are prone to poor healing, with difficulties reported for surgical repair [19,20]. Tensile stress may be an important factor for regeneration of insertion and tendon to bone healing during conservative treatments and/or surgical treatments such as ACL reconstructions and rotator cuff repair and so on. An experiment to add exogenous tensile stress after injury and surgery of tendon/ligament insertion is necessary. In bone lengthening using external fixation, insertions of tendon and ligament can extend during gradual elongation more than 4 weeks after the operation.

The limitations of this study were the use of a small number of animals for each group. Moreover, we investigated only the short-term results of elongation and used only one elongation speed in the study. Therefore, it is necessary to conduct long-term experiments exceeding 4 weeks using an improved external fixation device that can vary the elongation speed. Future experiments should also aim to determine the histopathological differences between the uncalcified fibrocartilage and the calcified fibrocartilage at the insertion site. Not only gradual elongation but also primary tension at Time 0 might occur as a proinflammatory response. Therefore, an experiment to start gradual elongation several weeks after an operation is necessary for reducing the effects of the primary tension and surgical invasions. It is necessary to clarify which GAG chains are produced under tensile stress. In this study, we used skeletally immature rabbits. The growth plate and the PT insertion may be simultaneously extended under gradual elongation. Therefore, it is necessary to investigate experiments using skeletally mature rabbits. Finally, as it is currently unclear what signalling processes are used to create these changes in the cells, it will be important to elucidate the detailed of signalling cascades in future experiments.

## 4. Materials and Methods

### 4.1. Animal Experiments

**Figure 8:**
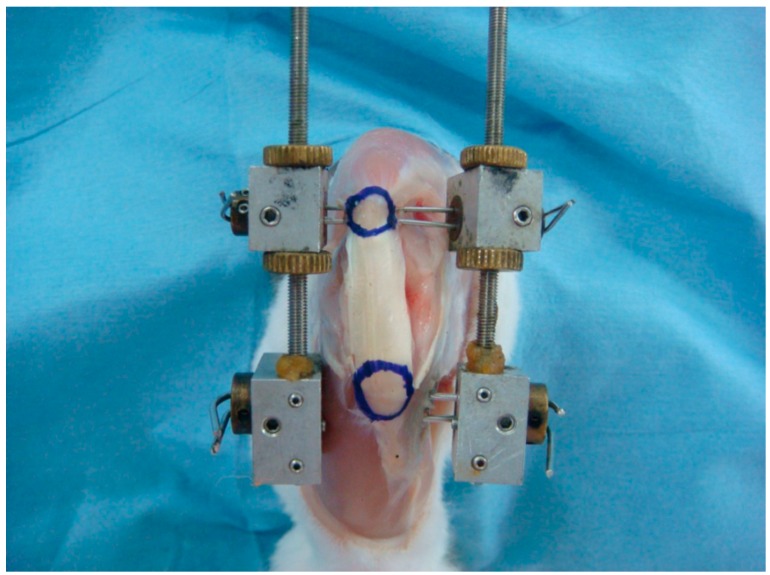
Position of the external fixation device in the right knee of a rabbit. Two stainless steel wires were inserted into the patella perpendicular to the patella-tendon axis, and another two stainless steel wires into the tibial tubercle perpendicular to the tibial axis. The external fixation device was positioned along both sides of the right knee. The upper blue circle indicates the patella; the lower blue circle indicates the tibial tubercle.

Fifty-four skeletally immature male Japanese white rabbits (weight range: 2.5–3.0 kg, 13 weeks of age) were used in this study. The experimental and housing conditions for the rabbits were the same as in our previous studies [6,8,9], and histomorphological analyses were performed by experienced technicians. The rabbits were maintained in accordance with the guidelines of the Ethical Committee of the University of Tsukuba and the National Institute of Health guidelines for the care and use of laboratory animals (NIH Pub. No. 85-23 Rev. 1985). The rabbits were divided into two groups (elongation group; *n* = 24, sham group; *n* = 24). After an intravenous injection of barbiturate (40 mg/kg body weight), an anterior skin incision was made over the right knee. The medial and lateral retinacula were incised longitudinally along both edges of the patellar tendon. Two stainless steel wires (0.8 mm in diameter) were inserted into the patella perpendicular to the PT axis, and another two stainless steel wires (1.2 mm in diameter) were inserted into the tibial tubercle perpendicular to the tibial axis. An external fixation device, manufactured by our group, was then installed along both sides of the right knee (Figure 8), and the wound was closed. We added primary tension so that PTs did not slack at Time 0. For this apparatus, each rotation of the dial induced an extension between the interval of the patella and the tibial tubercle of 0.5 mm. Elongation was commenced on day 1 after surgery and continued for 4 weeks at a rate of 0.5 mm/day. The same surgical procedure was performed on the animals of the sham group, with implantation of 4 stainless steel wires, without installation of the external fixation device. At 1, 2 and 4 weeks post-operatively, eight animals in each group were euthanized with deep anaesthesia to analyse the changes induced by the variations in elongation. We excluded one specimen of 2 weeks in the elongation group, because of infection. In this study, we did not do an experiment over 4 weeks gradual elongation, because of the structural problems of our model of external fixation and in consideration of pin site infection. Additionally, we used normal PT insertions (intact PT group) as reference value (weight range: 2.5–3.0 kg, 15–17 weeks of age, *n* = 6).

### 4.2. Histology, Immunohistochemical Staining and Histomorphological Analysis

Patella-tendon (PT) complexes were obtained from the hind limb of each animal as the direction of the structure alignment (bone-cartilage layer-PT) is parallel to the direction of the elongation. Specimens were trimmed to determine the sagittal plane of the central region of the insertion site and then fixed in 10% paraformaldehyde (pH 7.4) for 1 week. After fixation, all specimens were decalcified using 10% EDTA (pH 7.4) and embedded in paraffin. For each specimen, 5-μm-thick serial sections of the sagittal plane of the insertion site were stained with haematoxylin-eosin (HE, Sakura Finetek Japan Co., Ltd., Tokyo, Japan), according to standard laboratory protocols. Sections were then stained as outlined.

TUNEL staining was carried out in accordance with the instructions included in the Apoptag^®^ Plus Peroxidase In Situ Apoptosis Detection kit (Merck Millipore, Billerica, MA, USA), with the exception that we performed haematoxylin counterstaining. Briefly, sections were incubated in an equilibrium buffer for 10 min at room temperature. Sections were then incubated with terminal deoxynucleotidyl transferase (TdT) and digoxigenin-labelled nucleotides for 60 min at 37 °C in a humid chamber, followed by peroxidase-conjugated digoxigenin antibody for 30 min at room temperature in the humid chamber. The immunoreaction product was developed in diaminobenzidine, and the sections were counterstained with Mayer’s haematoxylin for 30 s. TUNEL-positive nuclei stained dark brown and TUNEL-negative nuclei stained blue [5,6,7,8,9].

Proliferating cell nuclear antigen (PCNA) immunostaining was performed in accordance with the manufacturer’s instructions (Histofine^®^ SAB-PO (M) kit; Nichirei Biosciences Inc., Tokyo, Japan). Deparaffinised sections were rinsed in PBS for 5 min and then immersed in 3% hydrogen peroxidase (H_2_O_2_) in methanol for 10 min to block endogenous peroxidase. Slides were then rinsed in PBS for 5 min, pre-blocked with a solution of 10% normal rabbit serum at room temperature for 10 min, and then incubated for 12 h at 4 °C with a monoclonal antibody to PCNA (PC-10, Code No. M0879, DAKO, Glostrup, Denmark) diluted 1:100. As a negative control, Antibody Diluent (Code No. S0809, DAKO, Carpinteria, CA, USA) was used in place of the primary antibody [7,9].

Histomorphometrical analyses were as previously described [6,7,8,9]. Since the tidemark was not clear by HE staining and Safranin-O staining, we used Weigert’s iron hematoxylin solution in Safranin-O staining for staining the tidemark. We could distinguish uncalcified fibrocartilage layer and calcified fibrocartilage layer by Safranin-O staining. However, the use of Weigert’s iron hematoxylin solution in TUNEL and PCNA staining adversely affected reactions of nuclear staining. Therefore, we evaluated two of the cartilage layers (uncalcified fibrocartilage layer and calcified fibrocartilage layer) as one cartilage layer in TUNEL and PCNA staining. The sections were examined under a light microscope (BX-51, Olympus Optical Co., Ltd., Tokyo, Japan) equipped with a CCD camera system (DP50, Olympus, Tokyo, Japan). The cartilage layer was distinguished between ligaments in terms of cell shape and the aspect of the extracellular matrix by HE staining. We measured the area of the Safranin-O-stained GAG layer in the uncalcified fibrocartilage layer and calcified fibrocartilage layer between the ligament and lamellar bone by Safranin-O staining. Using Motic Images Plus 2.1S software (Shimadzu, Tokyo, Japan), the stained GAGs areas in the uncalcified fibrocartilage layer and calcified fibrocartilage layer were determined. Each stained GAG area was divided by the width of the PT insertion, and the values obtained were defined as the average thicknesses of the stained GAGs areas in the uncalcified fibrocartilage layer and calcified fibrocartilage layer. The average percentages of TUNEL-positive chondrocytes and PCNA-positive chondrocytes were calculated from the positive cell counts for each marker divided by the total number of chondrocytes in the cartilage layer. The measurements of the cells and GAGs were blinded for each examiner. 

### 4.3. Statistical Analysis

The obtained histomorphological data were compared using Student’s *t*-test, with significance set at *p* < 0.05.

## 5. Conclusions

Gradual elongation of PT insertion significantly affected the increase in the average thicknesses of the stained GAGs areas in the cartilage layer especially in the uncalcified fibrocartilage layer at 4 weeks in rabbits. Clinically, insertions of tendon and ligament can extend during gradual elongation using external fixation more than 4 weeks after the operation.
